# Brine shrimp cytotoxicity of crude methanol extract and antispasmodic activity of α-amyrin acetate from *Tylophora hirsuta* Wall

**DOI:** 10.1186/1472-6882-13-135

**Published:** 2013-06-17

**Authors:** Niaz Ali

**Affiliations:** 1 Department of Pharmacology, Institute of Basic Medical Sciences, Khyber Medical University, Peshawar, KPK, Pakistan

## Abstract

**Background:**

We have previously reported that aerial parts of *Tylophora hirsuta* have antispasmodic profile. The current work is an attempt for isolation of pharmacologically active compound(s) that contribute for its antispasmodic activity.

**Methods:**

Preliminary phytochemical screening for crude methanol extract of *Tylophora hirsuta* (Th.Cr) is performed. Brine shrimp cytotoxicity of crude methanol extract is performed. Column chromatography was used for isolation of compounds. Mass spectroscopy, H^1^ NMR and C^13^ NMR were used for structural determination of compounds. α-amyrin acetate was tried for possible spasmolytic activity in rabbit’s jejunal preparations and KCl-induced contractions.

**Results:**

Th.Cr tested positive for saponins, alkaloids, flavonoids and terpenoids. Compound 1 was isolated as α-amyrin acetate. Compound 2 was heptaeicosanol. Crude methanol extract tested positive for brine shrimp cytotoxicity with LC_50_ 492.33± 8.08 mg/ml. Compound 1 tested positive for antispasmodic activity on spontaneous rabbits’ jejunum preparations with EC_50_ (60 ± 2) × 10^-5^M. The compound also tested positive on KCl induced contractions with EC_50_ (72 ± 3) × 10^-5^M.

**Conclusions:**

The present work confirms that α-amyrin acetate is has antispasmodic profile and the relaxant effect may be attributed to α-amyrin acetate which is a major compound.

## Background

*Tylophora hirsuta* belongs to family asclepiadaceae (common name: Tylophora) which is of great medicinal importance [1,2]. It is a shrub that belongs to family asclepiadaceae [1]. Asclepiadaceae is mainly distributed in the tropical and subtropical regions of the world, which have 175–180 genera and 2200 species as reported by Yasin J. Nasir (1983) [1]. Family asclepiadaceae has great ethnobotanical importance in the treatment of different diseases [3]. For example, *Dregea volubilis* is used in the treatment of snake bites and skin infections like boils and cellulites. *Oxystelma racamone* is used in the treatment of sore throat and jaundice. While *Pentatropis spiralis* is emetic and astringent. It is also used in the treatment of gonorrhoea. *Ceropegia bulbosa* is used in the treatment of digestive disorders, and tonic as well. Similarly, *Pergularia daemia* is used in the treatment of asthma, diarrhoea, amenorrhea and other gastro intestinal disorders. Genus asclepiadaceae, in Pakistan, is represented mainly by two species i.e. *Tylophora hirsuta* and *Tylophora tenerrima*. *Tylophora* is of great medicinal importance. It is used, traditionally, for the treatment of asthma, diarrhoea, rheumatism, management of hypertension and other allergic conditions [4]. It is mentioned in Bengal Pharmacopoeia since 1884. Other reported activities are anti-allergic and anti-arthritic [5,6]. Cytotoxic activities of *Tylophora asthmatica* has been reported in male rats [7]. So far, reported alkaloids from *Tylophora hirsuta* are 13 a-methylo-hirsutine, tylohersutinine, 13a-methyltylo-hirsutinidine and tylohirsutinidine [8,9]. In 1987, Bhutani et al. reported its anti-amoebic activity [10]. Other reported constituents from the aerial parts of the *Tylophora hirsuta* are Gymnorhizol, and β-sitosterol [11]. We have reported that crude methanol extract of *Tylophora hirsuta* has antispasmodic activity where we emphasized for isolation of pharmacologically active compounds. The current work is an attempt for isolation of pharmacologically active compound(s). Brine shrimp cytotoxicity of crude methanol extract is also performed.

## Methods

### Collection, authentication and extraction of plant materials

The aerial parts of the plant were collected from the nearby hills of University of Malakand in the year 2006. The plant was identified by professor Dr. Jehandar Shah, Vice Chancellor University of Malakand. A voucher specimen (Th.01) was submitted to the herbarium of University of Malakand. The materials were subjected to shade drying. They were crushed and pulverized to fine powder. 7.5 kg of dried powdered materials were macerated with commercial grade methanol (80%) for 15 days. The materials were filtered. The filtrates were concentrated under reduced pressure using a rotary evaporator till we obtained 1200 grams of brownish crude extract (free of solvent).

### Preliminary phytochemical screenings

Preliminary phytochemical tests were performed for the presence of alkaloids, flavonoids, tannins, saponins, glycosides, terpenoids, sterols and carbohydrates [12-14].

### Solvents and chemicals

Commercial grade solvents were double distilled, which were used in the experiments. Analytical grade chemicals (Merck) were used in the experiments. All solutions were made on the same day of experiments.

### Drugs and animals

Acetylcholine was purchased from Poole chemicals, UK. Rest of the chemicals were purchased from Merck. Rabbits of either sex (weighing 1.5-2.5 kg) were purchased from the local market. They were housed in the animal house of University of Malakand. They were dealt as per “Animals Byelaws 2008 of the University of Malakand”. Ethical Committee of Department of Pharmacy, University of Malakand approved the experimental protocols (case no: E-03-7).

### Spectroscopy

Hitachi U-3200 Spectrophotometer was used for the determination of UV spectra. Infrared (IR) spectra were measured as a potassium bromide (KBr) pellet or in chloroform on JASCO 302-A Infrared Spectrometer. Low-resolution electron impact mass spectra were recorded on a MAT 311A mass spectrophotometer or Finnigan MAT 312 double-focusing mass spectrophotometer, coupled with PDP 11/34 computer system. HREIMS measurements and Fast Atom Bombardment mass measurement were determined on Jeol JMS HX 110 mass spectrometer. ^1^H-NMR spectra were recorded at 300 MHz on Bruker AM-300 using TMS as an internal reference.

### Isolation of compounds

Column chromatography (CC) was carried out on silica gel (Si 60, 70–230 mesh, E. Merck) as stationary phase. Organic solvents like *n*-hexane, chloroform, ethyl acetate, *n*-butanol were used as mobile phase(s) as mixture for separation of compounds. For the purification of fractionated extracts, flash column chromatography (FCC) was performed on Eyela Flash Chromatograph model EF-10, using silica gel (Si 60, 230–400 mesh, E. Merck grade) as adsorbent. Pre-coated silica gel GF_254_ preparative plates (E. Merck) of 20×20 and 0.5 mm thickness were used for preparative layer chromatography. Purity of the samples was also checked on the same pre-coated aluminium cards using suitable spray agent.

### Spray reagents for visualization of spot(s)/compound(s)

Saturated solution of Ceric Sulphate in 65% sulphuric acid [15] is used to locate the substances on TLC plates. On heating terpenoids gave pink color.

### Fractionation for isolation

1000 grams of crude methanolic extract was suspended in 500 ml distilled water. It was fractionated with *n*-hexane (3 × 450 mL), chloroform (3 × 450 mL), ethyl acetate (3 × 450 mL) and *n*-butanol (3 × 450 mL) to yield *n*- hexane (500 g), chloroform (45 g), ethyl acetate (20 g), *n*-butanol (5 g) and aqueous (250 g) fractions, respectively. 100 g of crude methanolic extract was reserved for other pharmacological screenings.

Solvent system was developed using ethyl acetate: *n*-hexane mixture with increasing order of polarity. The *n*-hexane fraction was loaded in a column as it showed antispasmodic activity (data not shown). The contents were eluted with increasing order of polarity: *n*-hexane: ethyl acetate solvent system i.e. 100% *n*-hexane, 2% ethyl acetate, 4% ethyl acetate, 6% ethyl acetate till 100% ethyl acetate was passed through the column.

Compound **1** was isolated at 3.5% ethyl acetate: *n*-hexane solvent system in a separate pencil column.

Compound **2** was obtained from *n*-hexane fraction using *n*-hexane: ethyl acetate (11.5:1) as solvent system.

### Data recording for effects on spontaneous rabbits’ jejunal preparations

Data was recorded with help of force Transducer (Model No: MLT 0210/A Pan Lab S.I.) connected with Power lab (Model No: 4/25 T) ADInstruments, Australia. Bridge Pod Amplifier connected with the Power lab was used for amplification of the intestinal responses. Other setting parameters were in range of 20 mv, Low pass 5 Hz × 10 gain (input 1) and @ 40/S.

### Interpretation of data and statistical analysis

Chart 5, supplied with the Power Lab, was used to interpret the data. Statistical analysis was performed at 95% confidence interval. *P* value equal to or less than 0.05 was considered as significant. Graph Pad prism was used to calculate mean, SEM and draw the curves for EC_50_ shift.

### Brine shrimp cytotoxicity

Brine shrimp eggs were hatched. 10 shrimps, 5 ml sea water and extract at concentrations of 10, 100 and 1000 ppm were added to a vial. After incubation of 24 hours on room temperature, brine shrimp cytotoxicity was performed. Numbers of shrimps survived were counted. Per cent cytotoxicity was determined [16].

### Effects on spontaneous rabbits’ jejunum preparations and KCl induced contractions

Rabbits of either sex (weight 1.9 - 2.5 kg) were subjected to cervical dislocation. Their abdomens were opened. Pieces of jejunums were removed and kept in Tyrode’s solution aerated with carbogen gas (95% oxygen: 5 carbon dioxide mixture). Mesentery was removed from the tissues. Concentration of constituents of used in Tyrode’s solution were (mM): KCl 2.68, NaCl 136.9, MgCl_2_ 1.05, NaHCO_3_ 11.90, NaH_2_ PO_4_ 0.42, CaCl_2_ 1.8 and glucose 5.55. Preparations of about 1.3 - 1.5 cm lengths were mounted in 10 ml tissue bath containing Tyrode’s solution. Temperature was maintained as 37 ± 1°C. After stabilization, α-amyrin acetate was applied in molar concentrations 2.3 ×10^-5^- 243×10^-5^.

Similarly, sustained contractions were produced by 80 mM solution of KCl in the rabbits’ jejunum. α-amyrin acetate was also tested on rabbits jejunal preparation in same concentrations to explain its mode of action [17,18].

## Results

Upon preliminary phytochemical screenings, methanol extract tested positive for flavonoids, saponins, tannins, glycosides, terpenoids, sterols, phenols and carbohydrates.

### **Spectroscopy results for characterization of** α**- amyrin acetate**

**Physical data**: Colourless crystalline needles.

**Yield**: 6.5 g, 1.15%

**MP**: 243°C

**[α]D**: + 76° (c = 1.0, CHCl_3_)

**IR (KBr), ν**_**max**_: 1730 (C=O), 1640 (C=C), 1380 (CH_3_-), 1370 (CH_3_- / CH_3_CO), 1250 (−CH_2_-), 1030, 1000, 985, 960 (C-H) cm-1

^**1**^**H NMR**: (300 MHz, CDCl_3_): δ 0.79 (3H, *s*, H-28), 0.88 (12H, *s*, H-23, 24, 29, 30), 0.98 (3H, *s*, H-26), 1.01 (3H, *s*, H-25), 1,07 (3H, *s*, H-27), 2.05 (3H, *s*, OAc), 4.50 (1H, *dd*, *J* 9.7 Hz, H-3α), 5.12 (1H, *t*, *J*_*1*,*2*_ 3.6 Hz, *J*_*1*,*3*_ H-12)

**EI-MS**: (70 ev) *m*/*z* (rel. int): 468 [M]^+^, 453 (10), 408 + (20), 218 (100), 203 (10)

**Figure 1:**
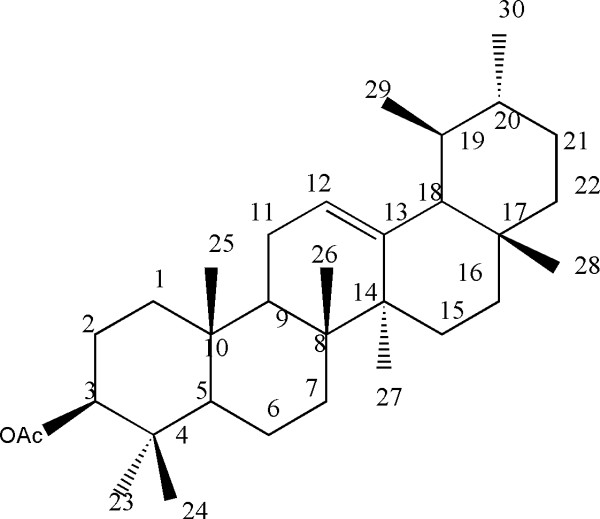
**Structural formula for α-amyrin acetate isolated from *Tylophora hirsuta*.**

^**13**^**C-NMR (CDCl**_**3**_**, 75 MHz):** Results of ^**13**^**C-NMR** is presented in Table 1 that represents the number of carbon atoms with their respective position in molecule. Structure resolved for is α- amyrin acetate expressed in Figure 1.

**Table 1:** ^**13**^**C-NMR (CDCl**_**3 **_**75 MHz) chemical shifts and multiplicities of ompound α- amyrin acetate**

**C. NO**	^**13**^**C- NMR**	**Multiplicity (DEPT)**
1	38.5	CH_2_
2	23.4	CH_2_
3	80.9	CH
4	37.7	C
5	55.3	CH
6	18.3	CH_2_
7	32.9	CH_2_
8	39.7	C
9	47.7	CH
10	36.8	C
11	22.8	CH_2_
12	124.2	CH
13	139.5	C
14	42.1	C
15	28.2	CH_2_
16	26.7	CH_2_
17	33.8	C
18	59.0	CH
19	39.7	CH
20	39.7	CH
21	31.3	CH_2_
22	41.6	CH_2_
23	28.1	CH_3_
24	15.8	CH_3_
25	14.2	CH_3_
26	16.8	CH_3_
27	17.6	CH_3_
28	28.8	CH_3_
29	23.3	CH_3_
30	21.4	CH_3_
COMe	21.5	CH_3_
CO	170.8	C

### Spectroscopy results for characterization of Heptaeicosanol (2)

**Physical data:** White solid from n-hexane fraction

**MP**: 81.6°C

**IR(KBR) ν**_**max**_: 3430 (H-bonded) and 720 [(CH_2_)n where n> 4] cm^-1^

^**1**^**HNMR (CDCl**_**3 **_**300 MHz)**: δ 0.88(3H, *J*= 7.1 Hz), 4.28 (2H, t, *J*=6.7 Hz, H-1), 1.28(m, CH2) _n._

**HREI-MS m/z:** 396.4329 (Calcd. For C_27_H_26_O; 396.4331)

**Figure 2:**
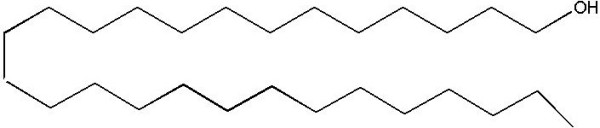
**Structural formula for Heptaeicosanol isolated from *Tylophora hirsuta*.**

Based on the spectroscopy, the structure resolved for compound 2 is expressed in Figure 2.

### Brine shrimps cytotoxicity of crude methanol extract

**Figure 3:**
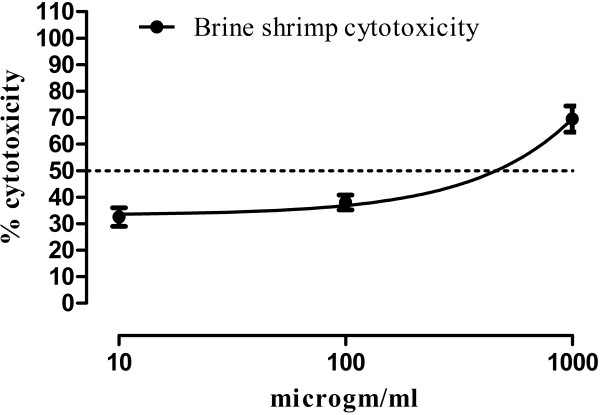
**Brine shrimp cytotoxicity of crude methanol extract of *Tylophora hirsuta*.**

Results of Brine shrimps cytotoxicity studies are summarized in Figure 3. The LC_50_ is 492.33± 8.08 (n=3) mg/ml for crude methanol extract.

### Effects on spontaneous rabbits’ jejunal preparations and KCl (80 mM)-induced contractions

**Figure 4:**
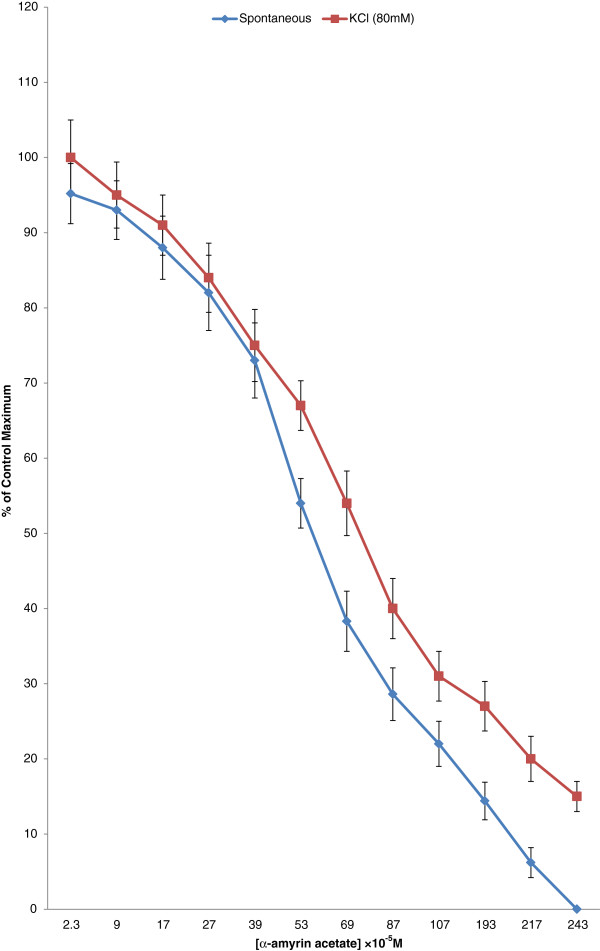
**The effects of α-amyrin acetate on spontaneous and KCl-induced contractions (values are mean ± SEM, n=4, *P *< 0.05).**

The relaxing effects of α-amyrin acetate on spontaneous rabbits’ jejunal preparations and KCl-induced contractions are expressed in Figure 4. Compound 1 produced relaxing effect as its concentration in the bath increased. Corresponding EC_50_ values for spontaneous and KCl- induced contractions are (60 ± 2) × 10^-5^ M and (72 ± 3) × 10^-5^ M.

## Discussion

The compound **1** was isolated as colourless needles from *n*-hexane fraction of methanolic extract of the aerial parts of *Tylophora hirsuta*.

The EI-MS of compound **1** showed the molecular ion peak at *m*/*z* 468, which was in agreement with the molecular formula C_32_H_52_O_2_ (calcd. *m*/*z* 468.) consistent with seven degrees of unsaturation. Besides molecular ion in the EI-MS, the mass spectrum showed characteristic fragmentation pattern of amyrin type skeleton with the double-bond at C-12 [19]. The IR spectrum displayed absorptions at 1730 and 1640 cm^-1^ due to the carbonyl and olefinic functions, respectively.

The study of ^1^H-NMR spectrum (CDCl_3_, 300 MHz) of compound **1** showed signals for methyls, methylenes and methin protons. In the up field region of the spectrum singlets each of three protons integration resonating at *δ* 0.97, 0.77, 0.94, 1.01, 1.13, 0.83 and 0.87 (6H) were assigned to tertiary methyls. In the downfield region of the spectrum a triplet of one proton integration resonating at *δ* 5.18 having a coupling constant J= 4.0 Hz, δ 2.05 was assigned to the olefinic protons H-12. Similarly a double doublet of one proton integration at α 3.21, with a coupling constant 10.2 and 4.3 Hz was assigned to H-3 α proton gemminal to acetate group. While a singlet of three proton integration resonating at *δ* 2.05 was assigned to methyl of acetate group attached at C-3.

The ^13^C-NMR spectrum (Broad band, DEPT) of compound 1 (Table 1) showed thirty two signals including nine methyls, nine methylenes, six methines and six quaternary carbons. The ^1^H- ^13^C correlations were determined by HMQC spectrum. In the ^13^C-NMR spectrum of compound-1, the up field resonance at *δ* 28.1, 15.8, 14.2, 16.8, 17.6, 28.8, 23.3 and 21.4 were due to the tertiary methyls while the signal at *δ* 21.5 was assigned to the carbon of methyl of acetate group. Similarly, in the down field region, the resonance at *δ* 170.8 and 124.2 were assigned to the carbonyl carbon and olefinic C-12. While the signal at *δ* 139.1 was due to the quaternary C-13 carbon.

The chemical shifts of these signals and other physical data were found identical to α-amyrin acetate (Figure 1) [20-23]. Compound **2** was obtained from the *n*-hexane fraction of the methanolic extract of aerial parts of *Tylophora hirsuta*. The structure of compound **2** was established by IR, mass and NMR spectroscopy. The molecular formula C_25_H_52_O was based on the HREIMS, FD and ^13^C-NMR spectra. HR-EIMS showed a molecular ion peak at *m*/*z* 368.01 (calcd. for C_25_H_52_O 368.04), suggesting the molecular formula C_25_H_52_O. The ^1^H –NMR spectrum displayed a triplet at δ 0.86 (*J*= 7.0 Hz) and a broad singlet at δ 1.21 typical of a straight chain hydrocarbon. It also showed signals at δ 3.85 (αH, H-1) respectively. The ^13^C-NMR spectrum (BB and DEPT) was also very informative in the structure elucidation of compound **2**. One oxygenated methylene resonated at δ 63.21 respectively. The signals observed between 29.0- 29.73 indicated the presence of a long chain hydrocarbon and the data correlated with reported literature [24]. The position of the hydroxyl group was also confirmed by the HMBC experiments (data not shown). Thus the structure of compound 2 was resolved as **Heptaeicosanol** (Figure 2).

The results of brine shrimps cytotoxicity study, LC_50_ is 492.33± 8.08 (n=3) mg/ml, reveal that the plant species can also be a potential source of cytotoxic compound(s).

As the plant is rich source of terpenes and terpenoids, hence our findings are in consistent with phytochemistry as α-amyrin acetate was the major compound isolated for the first time from *Tylophora hirsuta*. Being a major compound, the molecule was tested for possible relaxant activity on spontaneous rabbits’ jejunal preparations. It relaxed the spontaneous contractions and showed a concentration dependent relaxing effects. Since there are many mechanisms involved in the relaxing effects like relaxing effects either may be through calcium antagonistic action or through muscarinic receptors blocking mechanisms. There may be involvement of histaminergic receptors. Nevertheless, relaxing effects on KCl-induced contractions provides a rapid screening for possible relaxing mechanisms through voltage gated channels [25]. Voltage gated channels play a vital role in the regulation of peristaltic movements of the intestine as it helps in periodic depolarization and repolarization [26]. Since KCl induces contractions via calcium influx from extracellular fluid to intracellular medium, hence, relaxing effects on KCl-induced contractions are usually regarded as to follow calcium channel blocking mechanism [18]. Further work is required to elucidate possible mechanism by constructing calcium chloride curves and study its effects for possible relaxing mechanism. α-amyrin acetate can be a potential target for new drug development for management of hypertension and other cardiovascular disorders as calcium antagonists have anti-anginal, antihypertensive, and antiarrhythmic activity. The current work is further strengthening our previous reports about crude methanol extract of *Tylophora hirsuta* to have antihypertensive [27] and calcium channel blocking activity [4].

## Conclusions

α-amyrin acetate and Heptaeicosanol is reported for the first time from *Tylophora hirsuta*. The relaxing effect of *Tylophora hirsuta* may be attributed to α-amyrin acetate a major compound from the plant that warrants further work for new drug development.

## Competing interests

The author has declared no competing interests.

## Pre-publication history

The pre-publication history for this paper can be accessed here:

http://www.biomedcentral.com/1472-6882/13/135/prepub
